# The bed nucleus of the stria terminalis as a neuromodulatory target for refractory epilepsy

**DOI:** 10.3389/fnetp.2026.1800427

**Published:** 2026-07-03

**Authors:** Jackson Murray, Eunyoung Hong, Sarah Mulloy, William Nobis

**Affiliations:** 1 Vanderbilt University Vanderbilt Brain Institute, Nashville, TN, United States; 2 Vanderbilt University Medical Center, Nashville, TN, United States

**Keywords:** bed nucleus of stria terminalis (BNST), epilepsy, neuromodulation, respiration, SUDEP (sudden unexpected death in epilepsy)

## Abstract

Refractory epilepsy remains a significant clinical challenge, affecting over a third of patients with epilepsy and drastically increasing their risk of sudden unexpected death in epilepsy (SUDEP). Furthermore, psychiatric comorbidities - such as depression and anxiety disorders - are highly prevalent among patients with epilepsy. As neuromodulatory therapeutic approaches evolve, it is paramount that novel targets for stimulation are explored that may concomitantly reduce seizure burden, ameliorate psychiatric comorbidities, and/or reduce SUDEP risk. Here, we review physiological factors that are thought to contribute to SUDEP and which may reduce risk of SUDEP if addressed therapeutically, then we describe how the bed nucleus of the stria terminalis (BNST) represents a forebrain limbic region that concurrently influences many of these physiological processes. We conclude by synthesizing recent findings regarding the BNST in patients and preclinical models of epilepsy and propose that this evidence positions the BNST as a promising extra-thalamic target for therapeutic neurostimulation in patients with refractory epilepsy.

## 1 Introduction

Epilepsy is a prevalent neurological disorder characterized by recurrent seizures and affects more than 50 million individuals worldwide (Feigin et al., 2025). Despite the availability of anti-seizure medications, over a third of patients have refractory seizures (Hakami, 2021). As a network-level disorder, epilepsy is often accompanied by psychiatric and cognitive comorbidities, which significantly impact a patient’s quality of life and may be exacerbated by refractory seizures (Taylor et al., 2011). Beyond these issues, refractory epilepsy drastically increases a patient’s risk of sudden unexpected death in epilepsy (SUDEP), further underscoring the necessity of alternative therapeutic options to control seizures or reduce SUDEP risk (Buchanan et al., 2023; Hakami, 2021).

SUDEP is defined as death in people with epilepsy that is not caused by injury, drowning, or other known causes and is the leading cause of death in patients with refractory epilepsy (Buchanan et al., 2023). Moreover, SUDEP represents a substantial public health burden as it is second only to stroke in potential life lost due to neurological disease (Thurman et al., 2014). While the precise mechanisms of SUDEP remain poorly understood, common risk factors and a consistent sequence of pathophysiological events have been described across many clinical and preclinical reports (Buchanan et al., 2023; Friedman, 2022; Monté et al., 2024; Ryvlin et al., 2013). Across these observations, postictal central apnea (cessation of breathing that is centrally-mediated) precedes bradycardia and subsequent cardiac arrest (Buchanan et al., 2023; Friedman, 2022; Johnson et al., 2021; Ryvlin et al., 2013). Additionally, patients and animal models exhibit interictal deficits in arousal and ventilatory responses, suggesting that repeated seizures may cause interictal dysfunction that could conceivably increase SUDEP risk (Englot et al., 2020; Sainju et al., 2019; Sivathamboo and Perucca, 2021). Therefore, interictal and postictal deficits in arousal and cardiorespiratory function appear to play central roles in the etiology of SUDEP.

Clinical approaches to pharmacoresistant epilepsy could decrease SUDEP risk by either reducing overall seizure burden, correcting interictal cardiorespiratory deficits, or improving postictal arousal/ventilatory responses. Resective surgery is the gold standard approach for refractory focal epilepsy however not all patients are appropriate candidates, and there exists conflicting evidence regarding the efficacy of surgery in significantly reducing SUDEP risk. Neuromodulatory techniques, including responsive neurostimulation (RNS) and deep brain stimulation (DBS), are becoming more common - particularly in those non-surgical cases - with active evolution in determining the best stimulation targets to impact seizures (de Oliveira et al., 2025). As these devices continue to evolve, it is critical to consider targets that could not only impact seizure susceptibility but also psychiatric comorbidities of epilepsy and SUDEP risk. In this review, we discuss how neuromodulatory targeting of the bed nucleus of the stria terminalis (BNST) could address refractory epilepsy and reduce SUDEP risk. We first synthesize clinical and preclinical literature regarding SUDEP mechanisms and known risk factors, then draw primarily from preclinical literature to provide an anatomical and functional primer on the BNST, highlighting its connections with hypothalamic and brainstem circuits. Finally, we review emerging findings about the BNST in epilepsy and discuss how targeting this forebrain structure may be a beneficial approach to modify seizures and/or decrease SUDEP risk.

## 2 Sudden unexpected death in epilepsy

SUDEP is the leading cause of death in patients with refractory epilepsy, resulting in approximately 3,000 deaths per year in the United States (Buchanan et al., 2023). As SUDEP is inherently unpredictable and largely unwitnessed, its precise pathophysiology remains elusive. However, observations in epilepsy monitoring units suggest that a majority of SUDEP events follow convulsive seizures - either generalized tonic-clonic (GTCs) or focal to bilateral tonic-clonic seizures - and are characterized by postictal central apnea that precedes asystole (Friedman, 2022; Ryvlin et al., 2013). In addition to these clinical observations, case-control studies have uncovered many risk factors for SUDEP, which consistently include having uncontrolled GTCs, having nocturnal seizures, and medical nonadherence (Friedman, 2022; Harden et al., 2017; Sveinsson et al., 2020). In this section, we briefly discuss leading hypotheses about SUDEP pathophysiology and touch on common risk factors. (for more comprehensive reviews about SUDEP, see (Buchanan et al., 2023; Jiang et al., 2025; Monté et al., 2024)).

### 2.1 Respiratory dysfunction

Peri-ictal respiratory complications are frequently observed in GTCs and focal seizures (Elisa et al., 2025; Lacuey et al., 2024; Micalizzi et al., 2022) and current research implicates post-convulsive central apnea as a key component of SUDEP pathophysiology (Friedman, 2022; Ryvlin et al., 2013). Ictal central apnea has widely been described in association with both GTCs and focal seizures, and is often suggestive of focal seizures of the temporal lobe (Lacuey et al., 2018; Micalizzi et al., 2021; 2022). While shorter ictal apneas are common and may not pose any serious risk, apneas of longer duration have a greater chance of causing hypoxemia (Elisa et al., 2025; Lacuey et al., 2018; Micalizzi et al., 2022). Conversely, post-convulsive central apneas are more rarely observed and may be associated with SUDEP risk (Serrand et al., 2023; Vilella et al., 2019). While it has been proposed that ictal central apnea and post-convulsive central apnea might be mechanistically distinct, new findings have revealed that post-convulsive central apneas appear to largely follow seizures with ictal apnea relative to those without, perhaps indicating some functional overlap (Meletti et al., 2025; Vilella et al., 2019). As postictal apnea seems to play a precipitative role in SUDEP, better understanding how seizures impinge upon respiratory function can greatly inform our mechanistic hypotheses.

The mechanisms of ictal central apnea are generally considered to depend upon seizure propagation to forebrain structures that project to medullary respiratory centers, while direct seizure spread to these centers and/or brainstem spreading depolarization is hypothesized to underlie post-convulsive central apnea. Early work demonstrated that electrical stimulation of temporal lobe structures can cause respiratory depression or apneas, thereby providing some of the first evidence that forebrain structures may critically regulate respiration (Bonvallet and Gary Bobo, 1972; Kaada and Jasper, 1952). More recently, clinical studies have extended this literature by studying patients undergoing stereoelectroencephalographic (SEEG) monitoring for epilepsy surgery. In these studies, localized electrical stimulation of the amygdala and/or hippocampus reliably produces apneas of which the patients are largely unaware and fail to report dyspnea (Dlouhy et al., 2015; Lacuey et al., 2017; Lacuey et al., 2019a). Furthermore, intracranial EEG monitoring in patients during seizures has shown that ictal central apnea, oxygen desaturation, and peri-ictal hypoxemia is associated with seizure spread to the amygdala (Dlouhy et al., 2015; Jung et al., 2022; Nobis et al., 2019). Moreover, increased amygdalar volume is observed in patients who exhibit postictal central apnea when compared to controls and patients without seizure-induced apneas (Lam et al., 2024; Meletti et al., 2025; Micalizzi et al., 2024; Zeicu et al., 2023). These findings indicate that the amygdala may play a pivotal role in peri-ictal apnea, likely through its direct and/or indirect connections with brainstem cardiorespiratory centers. In support of this notion, a decrease in functional magnetic resonance imaging (fMRI) blood-oxygen level dependent (BOLD) activity in the medulla and superior pons appears to coincide with apnea driven by amygdala stimulation (Harmata et al., 2023).

Respiratory drive is highly regulated by peripheral chemoreceptors in the carotid and aortic bodies and central chemoreceptors in the retrotrapezoid nucleus (RTN), raphe nuclei, nucleus of the solitary tract (NTS), and hypothalamus (Guyenet and Bayliss, 2015; Yackle and Do, 2025). Prolonged apneas occur postictally in observed SUDEP cases and are indicative of abnormal chemoreceptive drive, which stimulates respiration to correct alterations in carbon dioxide (CO_2_) or oxygen (O_2_) concentrations in the blood or cerebrospinal fluid (Dereli et al., 2025; Ryvlin et al., 2013). In fact, seizures result in significant increases in end-tidal CO_2_ and both focal seizures and GTCs cause prolonged blunting of the hypercapnic ventilatory response (HCVR) - a measure of CO_2_ sensitivity (Seyal et al., 2010; Teran et al., 2023).

Several structures and neuronal populations appear to be involved in seizure-induced apneas and postictal blunting of ventilatory responses. Volume loss in the periaqueductal gray (PAG), thalamus, hypothalamus, and medulla is associated with peri-ictal hypoxia, with PAG and thalamic alterations correlating with severity of hypoxia (Allen et al., 2020). With regards to neuronal populations, the serotonergic system has received much attention. Most serotonergic neurons are located in midline raphe nuclei where they are situated in close proximity to cerebral blood vessels and monitor changes in plasma CO_2_ levels and pH (Severson et al., 2003). In preclinical models, seizures inhibit a subset of medullary serotonergic neurons and pharmacologically enhancing serotonin release prevents seizure-induced respiratory arrest (Tupal and Faingold, 2019; Zhan et al., 2016). In patients, serotonin reuptake inhibitors may decrease the chance of ictal central apnea and reduce peri-ictal hypoxemia (Lacuey et al., 2019b). Furthermore, retrospective studies have revealed significant volume loss in medullary regions containing serotonergic neurons in patients who later died of SUDEP (Mueller et al., 2014; 2018), suggesting that alterations in serotonergic signaling may mediate ventilatory deficits. (For a comprehensive review of serotonergic signaling and SUDEP, see (Petrucci et al., 2020))

In addition to postictal deficits in ventilatory responses, patients and animal models with recurrent seizures display interictal blunting of ventilatory responses (Bhandare and Dale, 2023; Kuo et al., 2019; Sainju et al., 2019; Teran et al., 2023). This interictal hypoventilation may predispose some individuals to prolonged postictal respiratory deficits. In fact, patients who exhibit strong reductions in their interictal HCVR report less dyspnea during hypercapnia and have more severe postictal hypercapnia (Sainju et al., 2019). While the neural correlates of this interictal dysfunction remain unclear, a recent fMRI study demonstrated that patients with epilepsy display greater BOLD activation than controls in several chemosensing regions, including the dorsal raphe nuclei, lateral hypothalamus (LH), and PAG during interictal hypercapnia (Hampson et al., 2022). These findings suggest that severe blunting of interictal ventilatory responses and/or greater fMRI BOLD activity in chemosensing regions during hypercapnia may represent clinical risk markers for SUDEP and/or targets for risk reduction.

### 2.2 Cardiovascular dysfunction

Alongside respiratory alterations, seizures can cause a variety of cardiac abnormalities. Most seizures, including both GTCs and focal seizures, are accompanied by ictal tachycardia (Leutmezer et al., 2003). While potentially alarming, ventricular tachyarrhythmias are rarely associated with seizures or SUDEP. Indeed, a study of 193 patients with refractory epilepsy utilizing implantable loop recorders found no actionable rhythms aside from prolonged pauses in a small minority of patients (Serdyuk et al., 2021).

The role of ictal asystole (IA) in SUDEP has been a subject of debate (Benditt et al., 2015). Historically, the documentation of IA prompted immediate concern and aggressive intervention with invasive pacing, under the assumption that cardiac arrest is the primary driver of SUDEP (Chaila et al., 2010). This practice has been challenged by the continuing evidence that pacing may not confer any protective benefits, as SUDEP has been documented in patients with functioning pacemakers installed for IA (Bank et al., 2018). Large cohort studies support this. In a review of 157 patients with documented IA and refractory epilepsy there was no associated mortality (Tényi et al., 2017), leading the authors to argue for a very limited application of cardiac pacemakers in the treatment of IA. The controversy remains, as recent work suggests that autoimmune epilepsy that occurs with IA may carry risk of SUDEP (Vogrig et al., 2024). However, postictal bradycardia remains the cardiac marker most consistently associated with SUDEP, often observed following postictal apnea in monitored cases and leading to postictal asystole and death. Beyond acute peri-ictal changes, patients may develop an “epileptic heart,” characterized by a higher prevalence of structural cardiac disease, heart failure, and valvular disease (Mayer et al., 2024).

One proposed mechanism of postictal cardiorespiratory collapse is brainstem spreading depolarization (SD) - a self-regenerating wave of depolarization associated with excess glutamate release and increased extracellular potassium. In various genetic models with triggered and spontaneous seizures, SD in the medulla coincides with cardiorespiratory arrest (Aiba et al., 2016; Aiba and Noebels, 2015; Loonen et al., 2019). More specifically, in a Kv1.1 KO mouse line, cortically triggered seizures which led to SD in medullary areas analogous to the NTS elicited EEG suppression, apnea, bradycardia, and asystole, and these effects were recapitulated by local initiation of SD in the NTS (Aiba and Noebels, 2015). This literature highlights medullary regions, such as the NTS, that critically regulate cardiorespiratory function and may underlie peri-ictal dysfunction associated with SUDEP.

Finally, interictal cardiovascular comorbidities are highly prevalent in patients with epilepsy (Verrier et al., 2021). In a manner similar to interictal ventilatory dysfunction, interictal cardiac dysregulation may contribute to an individual’s risk of SUDEP. Patients with refractory epilepsy exhibit autonomic dysregulation such as attenuated heart-rate variability (HRV) - variability in the inter-beat interval as a measure of autonomic cardiac regulation - and reduced baroreflex sensitivity in the interictal phase (Ansakorpi et al., 2002; Athira et al., 2023; Baysal-Kirac et al., 2017; Sivathamboo et al., 2021; Sivathamboo and Perucca, 2021). Furthermore, retrospective studies have found that interictal HRV reduction is more pronounced in patients with epilepsy who later died of SUDEP than those who did not, suggesting that abnormal HRV may represent a clinical biomarker for SUDEP risk (Myers et al., 2018; Sivathamboo et al., 2021). Indeed, one study found that the degree of HRV reduction in patients was correlated with latency to SUDEP following the study (Sivathamboo et al., 2021).

### 2.3 Sleep and arousal

Almost 70% of SUDEP cases occur during sleep and having nocturnal seizures represents a clinical risk factor for SUDEP (Ali et al., 2017; Friedman, 2022; Lamberts et al., 2012; Ryvlin et al., 2013). While the nocturnality of SUDEP is incompletely understood, factors such as being in the absence of a witness and postictal deficits in arousal while lying prone in bed are believed to contribute (Ali et al., 2017; Purnell et al., 2018). In addition, circadian rhythmicity and sleep-wake states influence cardiorespiratory function and seizure susceptibility - with a strikingly low seizure probability associated with rapid eye movement (REM) sleep (Bagshaw et al., 2009; Bernard et al., 2025; Buchanan, 2013; Ng and Pavlova, 2013; Stephenson, 2007). Thus, better understanding the convergence of ictal and postictal cardiorespiratory dysfunction, sleep physiology, and arousal processes appears essential to elucidating the mechanisms of SUDEP.

Sleep dramatically alters autonomic physiology, in ways that may contribute to SUDEP risk. Respiratory drive is reduced, and hypercapnic and hypoxic ventilatory responses are attenuated during sleep (Newton et al., 2014). This reduced baseline responsivity to changes in CO_2_ and O_2_ may be compounded with ventilatory depression due to seizures. Indeed, when compared to seizures during wakefulness, nocturnal seizures are associated with more severe peri-ictal hypoxemia in patients (Latreille et al., 2017). Preclinical findings align with these clinical observations as seizures triggered during sleep result in greater postictal respiratory depression, more severe seizures, and a higher risk of fatality than seizures triggered during wakefulness (Hajek and Buchanan, 2016).

Sleep-related blunting of arousal may also be compounded with peri-ictal deficits in arousal. Focal seizures and GTCs can markedly impair arousal and consciousness ictally and postictally (Blumenfeld, 2021; Englot et al., 2010; 2020). Related to postictal blunting of arousal, diffuse postictal generalized EEG suppression (PGES) and postictal immobility are considered potential SUDEP risk factors, as both phenomena have been observed in SUDEP cases and may be associated with postictal respiratory dysfunction (Bruno et al., 2020; Kuo et al., 2016; Ryvlin et al., 2013; Seyal et al., 2013). Nocturnal seizures appear to be more frequently followed by PGES than seizures during wakefulness, suggesting that the sleep physiology may influence postictal markers of arousal (Latreille et al., 2017; Peng et al., 2017).

As postictal deficits in arousal appear to play a significant role in SUDEP, interictal dysfunction of central arousal processes may influence SUDEP risk. Evidence of interictal derangement of arousal processes has been reported from a host of recent clinical studies of patients with temporal lobe epilepsy (TLE). These studies report reductions in structural and functional connectivity between brainstem arousal centers and subcortical/cortical regions and interictal deficits in alertness/vigilance (Englot et al., 2017; 2018; 2020; H. F. J. González et al., 2023). Moreover, functional dysregulation of arousal networks appears to be associated with disease severity and may normalize following successful resective surgeries, thus implicating seizures as a causal factor (H. F. J. González et al., 2019; 2020; 2023). Sleep disorders are also frequently comorbid with epilepsy and patients often report excessive daytime sleepiness, consistent with impaired arousal (Bergmann et al., 2021; Safarpour Lima et al., 2021). While interictal deficits in arousal and SUDEP risk has yet to be precisely investigated, mouse models have shown higher risk of SUDEP during states of reduced arousal and brainstem atrophy that includes arousal centers was most pronounced in those who later died of SUDEP (Hajek and Buchanan, 2016; Mueller et al., 2014).

The mechanisms underlying the nocturnality of SUDEP remain poorly understood but are likely multifactorial. These factors include sleep-related blunting of arousal and autonomic functions, peri-ictal perturbations, as well as environmental factors like being alone and/or lying prone in bed. Sleep is a radical biological process governed by the circadian rhythmicity of many molecular and cellular processes and its ability to modulate seizure susceptibility is only beginning to be unraveled (Bernard et al., 2025). Current evidence suggests that ictal and postictal deficits in cardiorespiratory responsiveness and arousal may be more severe during sleep than during wakefulness, perhaps contributing to SUDEP risk. As sleep disturbances and excessive daytime sleepiness are frequently reported by patients, understanding how seizures disrupt sleep architecture might also reveal novel angles for precision therapeutics in patients prone to nocturnal seizures (Bergmann et al., 2021; Bernard et al., 2025).

### 2.4 Stress and seizures

Epilepsy significantly impacts the quality of life of patients due to seizure-related physical limitations and psychosocial factors such as mental illness and stigma (Karakis et al., 2023; Ridsdale et al., 2017; Sirven, 2015; Tombini et al., 2021). People with epilepsy have a significantly higher prevalence of mood disorders compared to those without epilepsy, with depression affecting up to 62% of patients (Strzelczyk et al., 2023; Kanner et al., 2002). The impact of stress and anxiety on seizures is multifaceted, as people living with epilepsy experience increased levels of stress and stress represents a common seizure precipitant in up to 85% of people with epilepsy (Catalán-Aguilar et al., 2025; Espinosa-Garcia et al., 2021; Lang et al., 2018; Temkin and Davis, 1984).

Although the increased occurrence of GTCs is a major risk factor for SUDEP (Friedman, 2022; Harden et al., 2017; Ryvlin et al., 2013), the significance of stress as a seizure trigger and SUDEP is poorly understood (Błaszczyk and Czuczwar, 2016; Lathers and Schraeder, 2006). Many clinical SUDEP studies do not report on quality of life and its relation to mortality (Maguire et al., 2020). However, there are studies that suggest an influence of stress on SUDEP and describe changes in emotional wellbeing prior to SUDEP (Earnest et al., 1992; Simeone et al., 2024). Social media engagement may be representative of stress levels, and one small cohort of patients exhibited increased Facebook usage and altered verbosity preceding SUDEP (Wood et al., 2022). Overall, stress-related risk factors in epilepsy remain an understudied area in the current literature, emphasizing the need to investigate the impact of stress on seizure susceptibility and SUDEP risk.

Stress leads to the activation of the hypothalamic-pituitary-adrenal (HPA) axis and release of the stress hormone cortisol (Ring, 2025). This process is altered in people with epilepsy, as they have increased basal cortisol levels (Cano-López and González-Bono, 2019) and increases in cortisol levels in the morning have been found to precede the occurrence of GTCs (Campen et al., 2015). Recent work indicates that altered HPA axis signaling may contribute to SUDEP, as people with epilepsy that died from possible SUDEP had significantly lower cortisol levels than people with epilepsy without SUDEP and healthy controls (Basu et al., 2024). Preclinical studies have attempted to further characterize this relationship, demonstrating that epileptic mice with a dysfunctional HPA axis display altered behavioral phenotypes and increased mortality (Basu et al., 2024; Saunders et al., 2025). However, there seems to be a sex-dependent response to stress in these mice, as males with a dysfunctional HPA axis had increased SUDEP but females with early life stress had decreased SUDEP risk (Basu et al., 2024; Coleman et al., 2025). Alongside the striking prevalence of psychiatric comorbidities, these findings heavily emphasize the need for continued research using various preclinical models of stress and epilepsy to understand its influence on the SUDEP pathophysiology and risk (Jones and O’Brien, 2013; Lathers and Schraeder, 2006; McKee and Privitera, 2017).

### 2.5 Interictal dysfunction and SUDEP risk

Patients with medically uncontrolled seizures, especially GTCs, are at a particularly high risk of SUDEP (Friedman, 2022; Ryvlin et al., 2013). While having recurrent GTCs substantially increases SUDEP risk, SUDEP probability does not appear to vary stochastically across seizures. Interictal dysfunction in cardiorespiratory function and arousal have been observed in both patients and preclinical models, and this dysfunction may be associated with the severity of postictal abnormalities and/or SUDEP risk (Mueller et al., 2018; Sainju et al., 2019; Sivathamboo et al., 2021; Sivathamboo and Perucca, 2021). Thus, interictal autonomic or arousal deficits may act to augment SUDEP risk. In addition to these interictal deficits, retrospective imaging studies of SUDEP cases have described many structural alterations which appear to influence SUDEP vulnerability (Allen et al., 2019; Mueller et al., 2018; Wandschneider et al., 2015). Perhaps unsurprisingly, these studies report alterations in subcortical and brainstem areas known to regulate respiration, autonomic function, and arousal. For example, increases in amygdalar and parahippocampal volume - structures that drive apnea upon stimulation - were observed in patients that later died of SUDEP (Allen et al., 2019; Wandschneider et al., 2015). Alongside these alterations, volume loss was reported in subcortical and brainstem areas which influence arousal and autonomic functions, including the PAG, posterior thalamus, raphe nuclei, and medullary autonomic nuclei (Allen et al., 2019; Mueller et al., 2018; Wandschneider et al., 2015). Coupling these structural findings with frequently observed interictal deficits both a) provides evidence that repeated seizures may progressively compromise respiratory and autonomic function through network-level perturbations and b) suggests that therapeutically normalizing interictal deficits may reduce an individual’s SUDEP risk.

### 2.6 SUDEP as an integrative phenomenon

**FIGURE 1:**
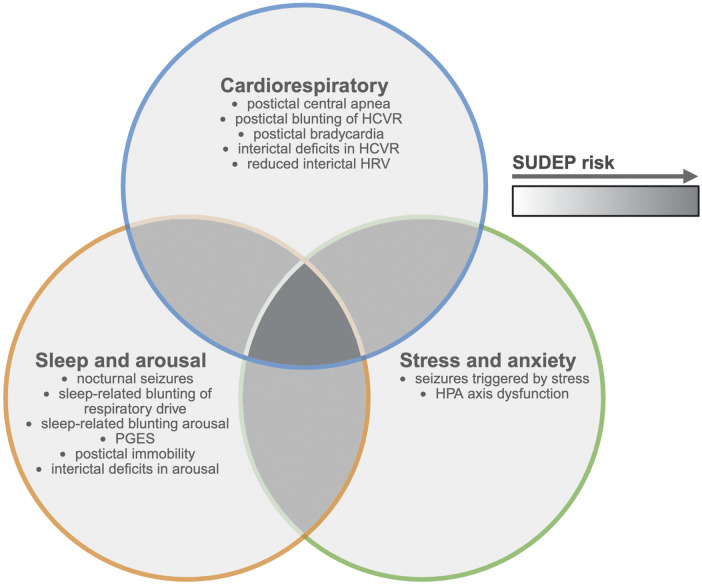
SUDEP risk as multifactorial, determined by the convergence of numerous physiological factors. Cardiorespiratory factors (blue circle) play a substantial role, particularly postictal central apnea and postictal blunting of ventilatory responses. Factors related to sleep and arousal (orange circle), such as the presence of nocturnal seizures and postictal blunting of arousal, may converge with cardiorespiratory deficits to increase SUDEP risk. Finally, psychiatric factors like stress and anxiety (green circle) may contribute to the convergence of these phenomena, thereby increasing SUDEP risk. SUDEP, sudden unexpected death in epilepsy; HCVR, hypercapnic ventilatory response; HRV, heart-rate variability; PGES, postictal generalized EEG suppression; HPA, hypothalamic-pituitary-adrenal. Created in BioRender. Murray J. (2026) https://BioRender.com/e53tbze.

Increasingly, the pathophysiology of SUDEP is considered to be a multifactorial collapse of postictal cardiorespiratory function which often follows a GTC during the night. Patients with refractory seizures are at a particularly high risk of SUDEP as having recurrent GTCs represents a major clinical risk factor. Converging evidence from clinical and preclinical studies suggest that repeated seizures may drive interictal abnormalities and network-level alterations involving key nuclei that control respiration, autonomic function, and arousal. Therefore, SUDEP represents an integrative phenomenon caused by the convergence of acute postictal deficits in cardiorespiratory drive and arousal, while interictal abnormalities and chronic neuropsychiatric comorbidities may augment the risk of a fatal alignment of these precipitating factors (Figure 1). Thus, we propose that forebrain hubs which concomitantly influence these many processes may represent promising targets for therapeutic approaches to refractory epilepsy and/or for SUDEP prevention.

## 3 Bed nucleus of the stria terminalis

The bed nucleus of the stria terminalis (BNST) is a multifaceted limbic region that displays remarkable continuity with medial regions of the amygdala, resulting in the anatomical concept of the ‘extended amygdala’ which includes the BNST and the central nucleus of the amygdala (CeA) (Alheid, 2003). The BNST contains diverse cell types that differ with respect to spatial organization, molecular signatures, synaptic input, physiological properties, projection sites, and functional roles, and these cells form extensive connections with cortical and subcortical networks to regulate stress, anxiety, reward-behaviors, feeding, arousal, and autonomic processes (Beyeler and Dabrowska, 2020; Kash et al., 2015; Shackman and Fox, 2016). In this section, we compile preclinical literature to describe efferent circuits that mediate physiological processes of concern in refractory epilepsy, focusing on stress and anxiety, arousal, and cardiorespiratory regulation. (For more detailed reviews of the BNST, see (Giardino and Pomrenze, 2021; Lebow and Chen, 2016))

### 3.1 Stress and anxiety

The BNST plays an important role in the behavioral and autonomic stress response by modulating both limbic and brainstem sites. In particular, the BNST forms connections with stress-activated regions such as the paraventricular nucleus of the hypothalamus (PVN) to modulate the HPA axis, as well as the LH, dorsal raphe nucleus (DRN) and ventral tegmental area (VTA) (Hammack et al., 2021; Kim et al., 2013; van de Poll et al., 2023). The BNST is heavily innervated by amygdalar subregions including the basolateral amygdala (BLA) and the CeA, however these structures serve to modulate different functions (Gungor and Paré, 2016; Walker et al., 2009). While the CeA is involved in short-term stress responses, the BNST is known to contribute to the anticipation of unpredictable stressors, which is related to chronic stress and anxiety (Davis et al., 2010; Goode et al., 2019; Lebow and Chen, 2016). For instance, targeted inhibition of gamma-aminobutyric acid (GABA) synthesis in the BNST increases anxiety-like behavior, and both chronic corticosterone (CORT) treatment and long-term social isolation have been shown to alter plasticity in the BNST (Conrad et al., 2011; Sajdyk et al., 2008).

The BNST contains one of the highest concentrations of extra-hypothalamic corticotropin-releasing factor (CRF) neurons in the brain (Dabrowska et al., 2016; Daniel et al., 2019). These BNST CRF neurons have been found to be involved in both anxiogenic and anxiolytic responses, signaling through the CRF type-1 receptor (CRFR1) or CRF type-2 receptor (CRFR2) (Gungor and Paré, 2016; Young and Tong, 2021). For instance, early-life maternal separation stress decreases CRFR1 receptor expression in BNST (Bonetti Bertagna et al., 2026). Furthermore, the electrophysiological and behavioral effects of early life stress and chronic variable mild stress can be rescued by CRFR1 antagonism (Hu et al., 2020a; Hu et al., 2020b).

As the primary driver of the HPA axis, the PVN contains many neurons that express CRF which are activated by stress and regulate HPA activity (Herman and Tasker, 2016). The PVN receives mainly GABAergic input, and some CRF-expressing and glutamatergic projections from multiple subregions of the BNST (Gungor and Paré, 2016; van de Poll et al., 2023). Damage to these subregions differentially affects PVN and HPA axis activity, suggesting that these subregions contribute to distinct components of the stress response (Herman and Tasker, 2016; Radley and Sawchenko, 2015). For instance, lesions to the posterior BNST increase circulating CORT levels and disinhibits the PVN in response to restraint stress (Choi et al., 2008). Specifically, targeted immunotoxin-mediated ablation of the fusiform and dorsomedial BNST GABAergic projections led to a similar increase in PVN and HPA activation in response to restraint stress (Radley et al., 2009). On the other hand, anterior BNST lesions led to decreased CORT levels and PVN activity in response to stress (Choi et al., 2007). Furthermore, BNST neurons that project to CRF-expressing neurons in the PVN were activated by both restraint and predator odor exposure, indicating that this circuit is engaged across diverse types of stressors (Lee et al., 2020). Moreover, the anterior nuclei of the BNST may have opposing effects on stress, as one study reported that the oval BNST promotes anxiety-like behavior, whereas the rest of the anterodorsal BNST may be involved in anxiolysis (Kim et al., 2013).

The DRN contains serotonergic neurons that are involved in anxiety-like behaviors and form reciprocal connections with the BNST (Hale et al., 2012; Nishitani et al., 2019; Sink et al., 2013; Sun et al., 2026; Zheng et al., 2024). Some studies indicate that anxiogenic stimuli activate cells in the BNST that project to the DRN, and the DRN may send serotonergic projections back to the BNST as a regulatory mechanism to reduce anxiety-like behavior (Hammack et al., 2009; Maier and Watkins, 2005). This is supported by the finding that CRFR2 inhibition in the DRN attenuates the anxiety-like behavioral response to inescapable tailshock stress (Hammack et al., 2003). On the other hand, serotonergic input to the BNST appears to engage an inhibitory microcircuit that decreases anxiolytic output to the LH and VTA (Marcinkiewcz et al., 2016).

The BNST and VTA also contain reciprocal projections, with the VTA sending GABAergic projections and the BNST sending mainly GABAergic and CRF-expressing projections (Koob, 2008; Marcinkiewcz et al., 2016; Root et al., 2020). Stress-related activation of BNST cells that project to VTA CRFR1-expressing cells leads to reinstatement of cocaine seeking in rodents (Briand et al., 2010; Vranjkovic et al., 2014). Although most of the BNST to VTA projections are GABAergic and mainly target non-dopaminergic neurons, there exists a smaller glutamatergic population that directly targets both GABAergic and dopaminergic neurons in the VTA (Jalabert et al., 2009; Kudo et al., 2012; Miura et al., 2023). This downstream targeting of GABAergic neurons in the VTA appears to play a dynamic role in the BNST’s coordination of anxiety. For instance, activation of glutamatergic neurons in the BNST that project to VTA GABA neurons promotes anxiety-like behaviors, whereas activation of GABAergic BNST neurons that project to GABAergic neurons in the VTA led to anxiolysis (Jennings et al., 2013b).

### 3.2 Sleep and arousal

Central regulation of sleep-wake architecture and arousal is highly interrelated with stress and anxiety processes, such that sleep disturbances are frequently comorbid with anxiety disorders (Antila et al., 2022; Chellappa and Aeschbach, 2022; Mao et al., 2024). As an example, prolonged sleep latency is a common manifestation during or after a stressful event in both humans and animal models and numerous studies have characterized the role of the amygdala/extended amygdala in this phenomenon (Kalmbach et al., 2018). Combined lesions of the CeA and the BNST in rats alleviates stress-induced sleep disturbances and chemogenetic inhibition of somatostatin neurons in the CeA ameliorates stress-induced changes in sleep latency (Cano et al., 2008; Yao et al., 2025). While these studies demonstrate that the extended amygdala plays a critical role in stress-related sleep disturbances, recent work has begun to characterize the role of the BNST in sleep and arousal more broadly.

Early recordings from single neurons in the BNST of cats revealed that roughly 70% of recorded neurons displayed state-dependent activity, with higher firing rates during wakefulness and REM sleep (Terreberry et al., 1995). More recently, cell-type specific fiber photometry in the BNST demonstrated that GABAergic neurons exhibit this same arousal-state-dependent activity, with greater activity during wakefulness and REM sleep than non-REM (Li et al., 2024). BNST GABAergic neurons are also significantly suppressed under isoflurane anesthesia, and photostimulation of these cells results in rapid transition to wakefulness from NREM sleep, REM sleep, or deep anesthesia (Kodani et al., 2017; Li et al., 2024). Relatedly, prepronociceptin-expressing GABAergic cells in the BNST rapidly modulate arousal in response to salient stimuli, and photostimulation of these cells increases pupil size and heart rate (Rodriguez-Romaguera et al., 2020). While the circuit-based mechanisms of these effects remain unclear, a thorough understanding likely involves projections from the BNST to numerous nuclei that control arousal and wakefulness, such as the VTA, LH, parabrachial nucleus (PBN), and the locus coeruleus (LC) (Giardino and Pomrenze, 2021; Stamatakis et al., 2014).

As mentioned above, the BNST sends GABAergic and glutamatergic projections to the VTA to influence reward-seeking behaviors, anxiety, and arousal (Dedic et al., 2018; Fellinger et al., 2021; Jennings et al., 2013b; Kudo et al., 2012; 2014; Rinker et al., 2017; Silberman et al., 2013). For example, stimulation of glutamatergic BNST terminals in the VTA drives aversive-like behaviors and anxiety, while stimulation of GABAergic BNST terminals in the VTA promotes reward-like behaviors and anxiolysis (Jennings et al., 2013b). Further, dopaminergic (DA) cells within the VTA are key regulators of wakefulness and arousal (Eban-Rothschild et al., 2018; Eban-Rothschild and De Lecea, 2017; Oishi and Lazarus, 2017). In fact, in a manner similar to BNST GABAergic neurons, VTA DA cells display arousal-state-dependent differences in activity and stimulation of DA neurons in the VTA drives wakefulness from sleep and anesthesia (Eban-Rothschild et al., 2016; Taylor et al., 2016). These data suggest that direct or indirect modulation of VTA dopaminergic signaling may serve as a circuit-based mechanism through which the BNST coordinates behavioral arousal. Indeed, wakefulness from sleep and anesthesia by photostimulation of BNST GABAergic neurons is recapitulated by photostimulation of BNST GABAergic terminals in the VTA (Li et al., 2024).

The LH regulates arousal and sleep-wake architecture, largely through two functionally opposed cell groups. These include neurons that express orexin and those that express melanin-concentrating hormone (MCH), which facilitate and inhibit wakefulness respectively (Arrigoni et al., 2019; Konadhode et al., 2015; Li et al., 2018; Mahoney et al., 2019). Additionally, the BNST sends GABAergic projections to the LH to influence stress responsivity, feeding, and arousal (Barbier et al., 2021; Giardino et al., 2018; Jennings et al., 2013a). These GABAergic projections broadly target glutamatergic neurons in the LH but contain subpopulations of CRF- and cholecystokinin-expressing cells that appear to differentially target specific LH populations, with CRF-expressing cells preferentially targeting orexin neurons (Giardino et al., 2018; Jennings et al., 2013a). Alongside targeting of orexinergic neurons, BNST GABAergic neurons exhibit direct inhibitory control over MCH neurons, providing a substrate through which the BNST may dynamically modulate the balance of orexinergic and MCH neuronal activity (González et al., 2016). In fact, acute restraint stress - a manipulation that robustly activates the BNST and is associated with arousal (Cullinan et al., 1995; Luchsinger et al., 2021; Xu et al., 2023) - is accompanied by a rapid, concurrent augmentation of orexinergic cell activity and reduction of MCH cell activity in the LH (González et al., 2016). Further, chemogenetic stimulation of BNST GABAergic neurons activates LH orexin neurons and results in sustained wakefulness that is suppressed by pretreatment with a dual orexin receptor antagonist (Kodani et al., 2017). Taken together, these findings suggest that the BNST may regulate arousal *via* dynamic modulation of orexinergic and MCH-expressing cells in the LH.

The PBN in the dorsolateral pons contains a large population of glutamatergic neurons that project widely to cortical and subcortical nodes to transmit information related to pain perception, influence feeding behaviors, regulate respiration, and stimulate behavioral arousal (Chen et al., 2022; Fuller et al., 2011; Kaur et al., 2017; Palmiter, 2018). In fact, the PBN is considered to be an essential component of the ascending arousal system, as saporin-based ablation of the PBN produces a coma-like state characterized by behavioral unresponsiveness (Fuller et al., 2011). PBN neuronal activity is also reduced under anesthesia and increases during emergence from anesthesia, and stimulation of the PBN promotes transitions to wakefulness (Luo et al., 2018; Muindi et al., 2016). Beyond emergence from anesthesia, chemogenetic activation of the PBN produces sustained wakefulness which is likely mediated by its projections to the basal forebrain and LH (Qiu et al., 2016).

The PBN and BNST form reciprocal connections, which are known to regulate responses to stress, feeding, affective states, and arousal (Flavin et al., 2014; Jaramillo et al., 2020; Luskin et al., 2021; Yan et al., 2024). Within the PBN, the neurons that express calcitonin gene-related peptide (CGRP) are particularly relevant. This population has been shown to regulate wakefulness, receive monosynaptic input from BNST glutamatergic and GABAergic terminals, and also project back to the BNST (Flavin et al., 2014; Kaur et al., 2017; Luskin et al., 2021; Yan et al., 2024). While the specific contribution of BNST projections to CGRP-expressing PBN neurons in wakefulness has yet to be uncovered, a recent paper demonstrated that glutamatergic PBN projections to the BNST regulate emergence from anesthesia (Yan et al., 2024). These findings indicate that the BNST and PBN may cooperate, both reciprocally and *via* projections to other nuclei, to modulate behavioral arousal.

The LC is the brain’s primary noradrenergic nucleus and sends broad projections from the brainstem to much of the brain to mediate arousal, attention, and cognition *via* norepinephrine (NE) release (Berridge and Waterhouse, 2003; Carter et al., 2010; Schwarz and Luo, 2015). Early electrical recordings of the LC in rats demonstrated that LC neurons display state-dependent activity, with highest activity during wakefulness and lowest activity during REM sleep (Aston-Jones and Bloom, 1981). Numerous more recent studies have further established the LC as a critical node for control behavioral arousal and sleep-wake dynamics (Antila et al., 2022; Carter et al., 2010; Hayat et al., 2020; Silverman et al., 2025). Specifically, optogenetic manipulation of the LC can bidirectionally control wakefulness, locomotion, and pupil dilation (Carter et al., 2010; Hayat et al., 2020). Interestingly, the effects of photostimulation of LC NE neurons are remarkably frequency- and duration-dependent. Acute stimulation rapidly induces arousal and sleep-to-wake transitions, but high-frequency or sustained stimulation results in behavioral arrests when awake and increased sleep pressure when asleep (Carter et al., 2010; Silverman et al., 2025). This may be indicative of functional fatigue of LC NE neurons and/or negative feedback mechanisms. The LC heavily innervates the BNST with noradrenergic fibers and the BNST sends projections to the LC, and their interaction is known to coordinate reward behaviors, fear, stress, and anxiety-like processes (Flavin and Winder, 2013; Kim et al., 2025; Reyes, 2025; Van Bockstaele et al., 1999). The extent to which BNST projections to the LC contribute to the LC’s control of arousal has yet to be thoroughly investigated. However, wakefulness driven by chemogenetic stimulation of BNST GABAergic neurons is accompanied by an increase in c-Fos expression in LC NE cells suggesting that activation of these neurons may be involved in the BNST’s control of behavioral arousal (Kodani et al., 2017).

### 3.3 Cardiorespiratory regulation

Alongside rapid modulation of arousal and wakefulness, the BNST is well-situated anatomically to influence cardiorespiratory function in response to stressful or emotionally valenced events or stimuli (Crestani et al., 2009; 2013; Dong and Swanson, 2004). Here, we compile literature regarding the BNST’s control of cardiovascular and respiratory physiology, and highlight projection targets that may underlie these effects.

A series of early publications provided some of the initial evidence that the BNST may critically modulate cardiovascular function (Ciriello and Janssen, 1993; Dunn and Williams, 1995; 1998; Li and Dampney, 1994; Potts et al., 1997). Reflecting the complexity of the BNST, stimulation of different subregions elicits different cardiovascular responses. Electrical or chemical stimulation of the medial regions of the BNST increases mean arterial pressure (MAP), while stimulation of lateral regions decreases MAP, and both pressor and depressor responses are produced *via* stimulation of the ventral BNST (Dunn and Williams, 1995; 1998). Different cardiovascular effects of stimulation were also reported along the anterior-posterior axis, such that changes in blood pressure and heart rate (HR) were strongest when the anterior BNST was stimulated - consistent with findings that the anterior BNST innervates many autonomic nuclei (Ciriello and Janssen, 1993; Dong and Swanson, 2004; Zhang et al., 2009). Further findings suggest that the BNST may regulate the baroreflex. Increases in blood pressure result in c-Fos expression in the BNST that is reduced by baroreceptor denervation, and reversible inactivation of synapses in the BNST with cobalt chloride enhances the bradycardiac response to increases in blood pressure without impacting the tachycardiac response to decreases in blood pressure (Crestani et al., 2006; Li and Dampney, 1994; Potts et al., 1997).

To understand the functional significance of the BNST’s regulation of cardiovascular responses, many studies have investigated these processes in the context of stress and exercise. Inhibition of local neurotransmission in the BNST roughly doubles the change in HR induced by acute restraint stress, without impacting blood pressure (Crestani et al., 2009). This effect is replicated by intra-BNST infusion of cannabidiol - an effect that is blocked by local infusion of a 5-HT_1A_ antagonist (Gomes et al., 2013). With respect to physical exercise, inhibition of neurotransmission in the BNST reduced both the pressor and tachycardiac response evoked by exercise, without affecting baseline MAP, HR, or baseline locomotion (Crestani et al., 2010). Subsequently, it was shown that ɑ1-and ɑ2-adrenergic receptors in the BNST differentially modulate exercise-induced cardiovascular responses. Selective antagonism of ɑ1-adrenergic receptors in the BNST enhanced the HR response to exercise without impacting the MAP response, while selective antagonism of ɑ2-adrenergic receptors reduced the exercise-induced increase in MAP without affecting the HR response (Alves et al., 2011). Taken together, these findings suggest that, while the BNST may not tonically influence cardiovascular function at rest, it represents a crucial forebrain structure involved in the regulation of cardiovascular responses.

Rapid adjustments in cardiovascular tone are often coupled to changes in respiratory drive, raising the possibility that the BNST regulates respiration and cardiovascular responses in parallel. Early electrical recordings in cats demonstrated that a proportion of cells in the BNST discharge rhythmically with the respiratory cycle, and these discharge relationships are dependent on sleep-wake states (Terreberry et al., 1995). Interestingly, the activity of a subset of neurons in the CeA display a similar state-dependent coupling with the respiratory cycle (Zhang et al., 1986). A more recent extension of these findings found that disinhibition (by local infusion of the GABA-A receptor antagonist bicuculline) of either the amygdala or the BNST resulted in robust, rapid, and dose-dependent increases in blood pressure, HR, and minute ventilation (Zhang et al., 2009). Thus, focal disinhibition of the BNST has been shown to evoke concurrent cardiovascular and respiratory responses, suggesting that this structure regulates both processes. Finally, photostimulation of the oval BNST and afferents in the surrounding anterodorsal subregion differentially influences respiratory rate, with stimulation of the oval BNST increasing respiratory rate and stimulation of the anterodorsal BNST reducing respiratory rate (Kim et al., 2013).

While much work has characterized the role of the BNST in cardiorespiratory regulation, a vast majority of these investigations have relied on focal manipulations so our circuit-based understanding about how the BNST elicits these responses remains obscure. Nevertheless, existing literature regarding central cardiorespiratory control and efferent BNST targets can inform hypotheses about underlying circuits.

Of particular interest are hypothalamic targets of the BNST, namely, the dorsomedial hypothalamus (DMH), PVN, and LH, which all contribute to cardiorespiratory regulation (Dampney, 2015; Lanzillo et al., 2021; Tenorio-Lopes and Kinkead, 2021). For example, inhibition of the DMH greatly attenuates neuroendocrine and respiratory responses to stress while activation of the DMH results in autonomic and respiratory effects that closely mimic those observed following acute stress (Bondarenko et al., 2015; McDowall et al., 2007; Stotz-Potter et al., 1996). And, in addition to regulating the neuroendocrine response to stress, the PVN plays a substantial role in respiratory regulation *via* its direct projections to medullary respiratory centers (Kc and Dick, 2010; Tenorio-Lopes and Kinkead, 2021). More specifically, vasopressin-expressing neurons in the PVN project to regions within the ventrolateral medulla where they influence cardiorespiratory drive and CRF cells in the PVN project to the NTS to coordinate the hypoxic ventilatory response (Kc et al., 2002; 2010; Ruyle et al., 2018; 2019; 2023). Lastly, LH orexin neurons facilitate ventilatory responses *via* direct projections to the NTS and by activating the aforementioned CRF-expressing PVN projection to the NTS (Ben Musa et al., 2024; 2026; Wang et al., 2024). Thus, as LH orexin neurons, PVN CRF neurons, and the DMH all receive input from the BNST, these sites represent efferent targets that may participate in the BNST’s influence of cardiorespiratory drive (Giardino et al., 2018; Huang et al., 2023; Lanzillo et al., 2021; Song et al., 2020). Supporting this idea, ablation of orexinergic cells in the LH significantly attenuates the increases in blood pressure, HR, and minute ventilation associated with disinhibition of the BNST (Zhang et al., 2009).

The PBN maintains a well-characterized role in cardiorespiratory regulation and the BNST and PBN form reciprocal connections that underlie stress, anxiety, and arousal (Davern, 2014; Flavin et al., 2014; Kaur et al., 2024; Kaur and Saper, 2019; Luskin et al., 2021; Yan et al., 2024). Intriguingly, broad photostimulation of the BNST drives an increase in respiratory rate but subregion-specific stimulation of excitatory afferents in the anterodorsal BNST results in a decrease in respiratory rate (Kim et al., 2013). This respiratory effect of anterodorsal BNST photomodulation is recapitulated by stimulation of BNST terminals in the PBN, but not replicated by stimulation of terminals in the LH or VTA (Kim et al., 2013). While it is unclear which neuronal populations in the PBN mediate this effect, the population that expresses CGRP represents a sound candidate as these cells are known to mediate respiration and hypercapnic arousal (Kaur et al., 2017; Luskin et al., 2021). With regards to respiratory rate, photostimulation of PBN CGRP neurons alone neatly replicates the effects of stimulating BNST terminals in the PBN (Bowen et al., 2020). The BNST also receives one of the densest PBN CGRP-expressing projections in the forebrain, rivaled only by projections to the CeA, and stimulation of PBN CGRP-expressing terminals in the BNST potently increases both heart rate and respiratory rate (Bowen et al., 2020; Pauli et al., 2022). Together, these findings suggest that this reciprocal circuit between the BNST and CGRP cells in the PBN may participate in cardiorespiratory regulation.

Caudal to the PBN, medullary cardiorespiratory centers may contribute to BNST-mediated changes in cardiorespiratory drive. For example, synaptic inhibition within the caudal ventrolateral medulla (CVLM) attenuates the cardiovascular effects of glutamate microinfusion in the BNST (Giancola et al., 1993). These data indicate that, while the BNST does not significantly project to the CVLM, the CVLM likely contributes to the BNST’s influence over cardiovascular function (Dong and Swanson, 2004). A medullary site that does receive direct projections from the BNST is the NTS, which is known to govern cardiorespiratory processes (Dong and Swanson, 2004; Zoccal et al., 2014). In the NTS, a subpopulation of neurons that express the transcription factor Phox2b play a critical role in orchestrating ventilatory responses and these cells receive direct input from neurons in the BNST (Fu et al., 2017; 2019; Shao et al., 2024). This may implicate NTS Phox2b neurons as important contributors to the BNST’s control of cardiorespiratory function.

## 4 The BNST in epilepsy

**FIGURE 2:**
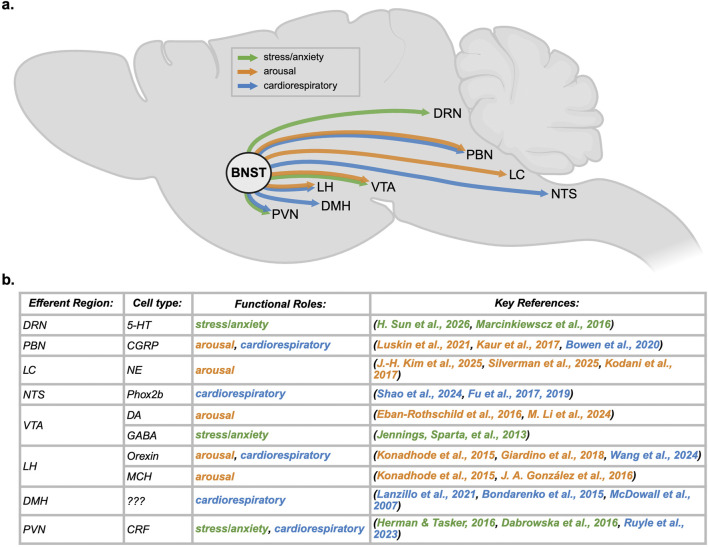
Efferent BNST targets with either known or hypothesized roles in modulating stress and anxiety, sleep and arousal, and cardiorespiratory regulation. **(a)** Subcortical and brainstem nuclei that receive direct projections from the BNST. These projections are proposed to contribute to the BNST’s regulation of stress and anxiety (green), sleep and arousal (orange), or cardiorespiratory regulation (blue). **(b)** Neuronal populations residing within these target sites with established roles in their respective processes and proposed to be either directly or indirectly modulated by BNST afferents. BNST, bed nucleus of the stria terminalis; PVN, paraventricular nucleus of the hypothalamus; DMH, dorsomedial hypothalamus; LH, lateral hypothalamus; VTA, ventral tegmental area; DRN, dorsal raphe nucleus; PBN, parabrachial nucleus; LC, locus coeruleus; NTS, nucleus of the solitary tract; 5-HT, serotonin; CGRP, calcitonin gene-related peptide; NE, norepinephrine; DA, dopamine; GABA, gamma-aminobutyric acid; MCH, melanin-concentrating hormone; CRF, corticotropin-releasing factor. Created in BioRender. Murray J. (2026) https://BioRender.com/uin6s16.

The BNST represents a forebrain limbic structure that receives input from hindbrain regions involved in ventilatory responses and arousal and projects to many hypothalamic and brainstem nuclei to coordinate stress responses, wakefulness and arousal, and cardiorespiratory function (Crestani et al., 2013; Flavin and Winder, 2013; Giardino and Pomrenze, 2021; Kim et al., 2013) (Figure 2). Therefore, as a critical integration hub for processes that are known to be dysregulated by epilepsy, the BNST may serve as a significant site of maladaptive plasticity across disease progression or as a promising site for therapeutic targeting. Indeed, recent findings from preclinical models and from clinical investigations provide converging evidence in support of this hypothesis.

Many recent studies have begun to explore the BNST in diverse preclinical models of epilepsy (Petrucci et al., 2024; Sun et al., 2024; Xia et al., 2022; Yan et al., 2021). DBA/1 mice exhibit convulsive seizures when exposed to a loud, broad tone and are commonly used to study SUDEP as these seizures are often followed by seizure-induced respiratory arrest, cardiac arrest, and death (Faingold et al., 2010; Marincovich et al., 2021; Schilling et al., 2019). In DBA/1 mice, convulsive seizures result in increased c-Fos expression in the BNST and disruption of synaptic transmission in the BNST significantly improves survival and reduces the occurrence of terminal ictal apneas (Xia et al., 2022). Additionally, in a mouse model of Dravet syndrome (DS) - a developmental and epileptic encephalopathy with an increased incidence of SUDEP - spontaneous seizures also increase c-Fos expression in the BNST (Yan et al., 2021). Furthermore, patch-clamp electrophysiological recordings from BNST cells in DS mice revealed that these cells exhibit altered spontaneous neurotransmission, reflecting an increase in excitability (Yan et al., 2021). Interestingly, circuit-specific electrophysiology demonstrated that BNST cells which project to the PBN are hypoexcitable in DS mice, perhaps contributing to interictal or postictal respiratory dysfunction and risk of sudden death (Yan et al., 2021). Taken together, these findings suggest that GTCs activate the BNST, the BNST may undergo intrinsic and circuit-level adaptations across disease progression, and modulation of the BNST might reduce seizure-induced respiratory dysfunction and SUDEP risk.

Beyond these direct investigations of the BNST, other preclinical studies have highlighted the BNST as a critical partner in broader circuits regulating epileptiform discharges and survival. Recently, it was demonstrated that stimulation of glutamatergic neurons in the posterior BLA can induce severe seizures in mice and the BNST mediates the excitability of these BLA cells *via* feedback inhibition (Sun et al., 2024). Specifically, diphtheria toxin-mediated deletion of GABAergic neurons in the BNST resulted in sporadic seizures and epileptiform discharges, but this effect was prevented by concurrent deletion of glutamatergic BLA neurons (Sun et al., 2024). Additionally, pre-seizure photostimulation of the DRN can reduce mortality in a maximal electroshock seizure mouse model and this site was shown to project to the BNST (Petrucci et al., 2024). This suggests that downstream modulation of the BNST might contribute to the observed decrease in mortality.

**FIGURE 3:**
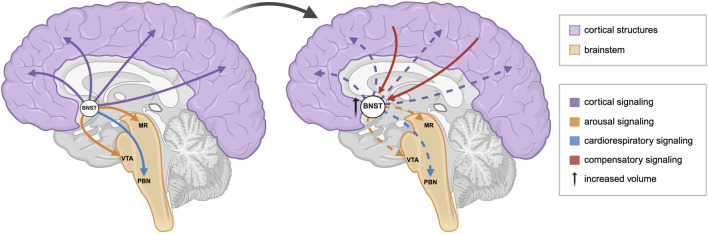
Alterations in BNST function across disease progression. The BNST projects widely across cortical and subcortical structures to coordinate arousal and cardiorespiratory processes (left). However, recent evidence from clinical and animal studies suggests that epilepsy drives structural and functional alterations involving the BNST (right). An increase in BNST volume (black arrow), and reduced functional connectivity with cortical structures (purple) and subcortical arousal-related structures (orange) is observed in patients with TLE. Additionally, preclinical studies have reported hypoexcitability of cells projecting from the BNST to the PBN (blue) in a genetic model of epilepsy. Collectively, these emerging findings indicate that the BNST is susceptible to maladaptive plasticity in epilepsy. BNST, bed nucleus of the stria terminalis; VTA, ventral tegmental area; PBN, parabrachial nucleus; MR, median raphe. Created in BioRender. Murray J. (2026) https://BioRender.com/y48ox9u.

Findings from structural and functional investigations of the BNST in patients with temporal lobe epilepsy (TLE) further indicate that this site is susceptible to maladaptive plasticity. Structurally, the BNST is significantly enlarged in patients with TLE (Dhaher et al., 2022). Intriguingly, patients without comorbid depression exhibit a unilateral enlargement of the BNST whereas a bilateral enlargement is observed in patients with comorbid depression, perhaps positioning the BNST as a viable therapeutic target for comorbid depression and epilepsy (Dhaher et al., 2022). Functionally, a recent study found that the BNST exhibits marked reductions in functional and effective connectivity with the whole brain - including temporal, thalamic, and brainstem networks - in patients with TLE (Reda et al., 2025). Moreover, patients displayed a decrease in BNST outflow to key arousal centers, namely, the VTA and median raphe (Reda et al., 2025). These findings indicate that TLE drives broad network reorganization that may ultimately compromise the BNST’s integrative role between brainstem cardiorespiratory centers and cortical arousal networks. Although these investigations did not directly study cardiorespiratory responses or SUDEP cases, the reported functional alterations could conceivably contribute to an individual’s risk for SUDEP. As the BNST is known to regulate arousal *via* GABAergic projections to the VTA, this reduced effective connectivity between the BNST and VTA may contribute to arousal deficits observed in patients with TLE (Englot et al., 2020; Li et al., 2024; Reda et al., 2025). Since the BNST coordinates broad arousal responses to hypercapnia, structural or functional alterations could contribute to blunted interictal or postictal arousal to hypercapnia (Taugher et al., 2014). Future work should characterize the functional and effective connectivity of the BNST in patients alongside arousal and/or cardiorespiratory assays to draw conclusions about the physiological relevance of this network reorganization and make causal inferences related to SUDEP risk. Nevertheless, the emerging focus on the BNST has resulted in critical insights that situate the BNST as a forebrain locus that is both acutely activated by seizures and chronically altered across disease progression (Figure 3).

## 5 Therapeutic potential

Resective surgery remains the gold standard treatment for medically refractory focal epilepsy (Harward et al., 2018). There is robust evidence that successful surgical resection drastically decreases SUDEP risk, likely by decreasing seizures (Casadei et al., 2020). However, more than half of patients with refractory epilepsy are either not candidates for resection due to overlapping eloquent cortex or multifocal onset, or they elect not to undergo open neurosurgery (Mandge et al., 2021). For this substantial population, neuromodulation has become a primary therapeutic avenue over the last decade. Current FDA-approved options include responsive neurostimulation (RNS) and deep brain stimulation (DBS). RNS utilizes a closed-loop approach where electrodes are placed directly in or near the ictal onset zone (IOZ) to deliver stimulation upon the detection of epileptiform activity. Conversely, DBS typically employs an open-loop, duty-cycle stimulation paradigm targeting deep thalamic nuclei, with the anterior nucleus of the thalamus (ANT) currently the only approved target. Both approaches reduce seizure frequency over time (Shi et al., 2025) and are associated with a reduction in SUDEP rates in longitudinal studies (Rheims et al., 2022; Salanova et al., 2021).

Despite these advances, significant limitations remain. A prominent side effect of ANT-DBS is the worsening or new onset of depression including suicidality (Doležalová et al., 2019), a concern that is particularly relevant given the high prevalence of baseline psychiatric comorbidities in this population. Furthermore, while RNS and DBS reduce overall seizure counts, stimulation often fails to abort seizures once they begin, and there is a paucity of data regarding how these therapies influence seizure severity or postictal physiology including cardiorespiratory decline, PGES, or arousal deficits.

As neuromodulatory strategies evolve to support more electrode leads and individualized programming, there is an opportunity to explore targets outside the traditional IOZ or thalamus. This includes targets that may not only influence seizure thresholds but also actively reduce comorbidities and SUDEP risk. The BNST represents a promising candidate for such precision therapeutics. Feasibility for BNST targeting is already established; it is currently being explored as a DBS target for treatment-refractory depression and obsessive-compulsive disorder (OCD), where it appears to be both safe and effective (Blomstedt et al., 2017; Goulas and Mavridis, 2025; Luyten et al., 2016). While they have not reported any cardiorespiratory effects of BNST stimulation in these studies, patients receiving BNST stimulation have noted excessive arousal and sleep disturbances (insomnia) (De et al., 2023; Naesström et al., 2021). While considered side effects in the context of psychiatric treatment, these phenomena suggest that stimulation of the BNST may normalize arousal deficits or excessive daytime sleepiness, both of which are observed in patients with TLE (Bergmann et al., 2021; Englot et al., 2020).

The BNST could be utilized as a dual-function target for next-generation responsive neurostimulation. Whereas chronic duty cycle stimulation of the BNST may produce hyperarousal, acute ictal and postictal stimulation of the BNST could serve as a “rescue” mechanism. Upon detection of a seizure, stimulation delivered to the BNST could activate its downstream brainstem effectors and enhance respiratory drive and promote arousal, thereby counteracting the potentially fatal postictal apnea and cerebral suppression associated with SUDEP. Stimulation of the BNST could also modulate the affective components of epilepsy, offering the positive mood stabilizing effects seen in the OCD trials, and offer an advantage over the depression-prone ANT target. Over time, normalizing activity in this hub could also stabilize interictal autonomic tone, potentially correcting the heart rate variability and chemoreception deficits observed in high-risk patients (Rao and Rolston, 2023).

## 6 Conclusions

Over a third of patients with epilepsy have refractory seizures, drastically impacting quality of life and increasing their risk of SUDEP (Buchanan et al., 2023; Hakami, 2021). In addition to surgical resection, neurostimulatory devices - such as DBS and RNS - are becoming more prevalent as a treatment for refractory epilepsy (de Oliveira et al., 2025). As these approaches become more refined and targeted, exploring targets that influence not only seizure burden but also psychiatric comorbidities and SUDEP risk will inform and improve therapeutic scope. Based on findings from clinical and preclinical studies, the etiology of SUDEP appears to depend on the convergence of postictal blunting of cardiorespiratory drive and arousal (Figure 1). Critically, interictal deficits in cardiorespiratory function or arousal and structural perturbations in the networks that mediate them may facilitate the fatal alignment of postictal deficits (Allen et al., 2019; Mueller et al., 2014; Sainju et al., 2019; Sivathamboo et al., 2021; Sivathamboo and Perucca, 2021). Thus, reducing seizure frequency, improving interictal abnormalities, or correcting postictal deficits could effectively reduce SUDEP risk.

Many nuclei that contribute to cardiorespiratory regulation or arousal are located in the brainstem, limiting the feasibility of neurostimulatory targeting. In this review, we describe how the BNST represents a forebrain structure that concomitantly influences cardiorespiratory drive and arousal (Figure 2). Moreover, the BNST is known to be involved in seizure-induced death in preclinical models and chronically altered in patients with TLE (Figure 3). Additionally, DBS in the BNST is already being explored as an approach to treatment-resistant OCD and depression (Goulas and Mavridis, 2025; Sobstyl et al., 2025). Therefore, the BNST is therapeutically accessible and may constitute a promising extra-thalamic site for the treatment of psychiatric comorbidities and/or the reduction of SUDEP risk in patients with refractory epilepsy.
